# Serious adverse event: late neurotropic disease associated with yellow fever vaccine

**DOI:** 10.31744/einstein_journal/2020RC5041

**Published:** 2020-02-13

**Authors:** Henrique Souza Barros de Oliveira, Patricia Padial de Araujo, Jamile Rafaela Poltronieri de Sousa, Ana Carolina Gariba Donis, Deise Moreira, Andrea Makssoudian

**Affiliations:** 1 Centro Universitário São Camilo São PauloSP Brazil Centro Universitário São Camilo, São Paulo, SP, Brazil.; 2 Hospital Geral de Carapicuíba CarapicuíbaSP Brazil Hospital Geral de Carapicuíba, Carapicuíba, SP, Brazil.

**Keywords:** Immunization, Yellow fever vaccine, Drug-related side effects and adverse reactions, Encephalomyelitis, acute disseminated, Yellow fever, Child health

## Abstract

The yellow fever is a systemic disease that was under control due to the effective campaigns against the vector and promotion of vaccines programs. However, since 1999, outbreaks appeared because of inefficient control of the vector, and led to the need of amplifying the immunization in large scale against the yellow fever virus, and consequently, raising the risk of adverse reactions to the vaccine. We report a case of previously healthy infant, who was referred to our care service, after 3 days with fever, chills, nausea and vomits, he received support therapy and was discharged from the hospital. After 24 hours of supportive measures, he was discharge. The patient returned to our service with general condition decline, strabismus, inability to control of cervical musculature and reduced force of the legs. The patient vaccine had received all vaccines from the calendar, and he was vaccinated for yellow fever 20 days before symptoms. During the hospitalization, liquor was collected, and ceftriaxone and aciclovir were administered. After negative cultures from the liquor, the antibiotics were suspended. The computed tomography of patient’s brain showed no alterations. Research for antibodies against yellow fever was requested, being positive for IgM in the liquor, and confirming the neurotropic disease associated with the yellow fever vaccine. On the fifth day of hospitalization, the patient showed improvement on the strabismus, cervical tonus, and musculature force. On the tenth day of hospitalization, patient showed complete improvement, and his laboratory exams no alterations. Subsequently, patient was discharged. The vaccine against yellow fever is safe, efficient and highly recommended, however it is not completely free from serious adverse reactions, including death.

## INTRODUCTION

Yellow fever (YF) is systemic disease with high morbimortality that is often found in tropical regions. YF is caused by the Flavivirus virus and transmitted by mosquitoes, such as *Aedes aegypti* (in urban cycle) and *Haemagogus* (in wild cycle).^1^ This disease was under control since 1940 because of the effective campaigns against *Aedes aegypti* and to the vaccination programs for those living at risky areas or people who traveled to such areas. However, new outbreaks appeared since 1999, because of inefficacy of policies to control the vector. From 2008 to 2018, the vector started to spread in Southeast and Central east regions of Brazil.^2^

Immunization in large scale against YF virus becomes a national need. This fact associated with own characteristics of vaccine against YF (VAYF), has led to important increase of risk of occurrence of adverse reactions (AR) to the vaccine.^3^

Because of the lack of data about AR associated with YF, we report a case of late neurotropic disease secondary to YF vaccine.

## CASE REPORT

This was a 9-month-old male infant, weighting 10.200g, who were previously healthy and who were from the municipality of Sao Paulo. Initially, the patient was admitted to the emergency room with a history of persistent fever (38°C), chill, nausea, and vomiting for 3 days. He received support therapy and was discharged. After 24 hours, the patient’s clinical picture evolved with worsening in his general status, strabismus, lack of control of cervical musculature and reduction of muscle strengths in lower limbs. At this time, the patient’s legal responsible sought to a referral hospital in the region.

The legal responsible mentioned that patient had no history of previous hospitalizations, use of medications, allergies, hereditary diseases, contact with ill individuals or any remarkable changes during the last months of life. In relation to immunization card, the patient had received all vaccines required to his age, however, 20 days prior to hospitalization, he had received VAYF. However, no reactions occurred before the vaccination.

During hospitalization process, supporting measures and complementary exams were requested ( Table 1 ). After biochemistry result of cerebrospinal fluid (CSF) test, an antibiotic therapy of large spectrum was initiated including ceftriaxone and acyclovir. After 48 hours of the introduction of antibiotic therapy, the result of CSF test was negative for bacteria, therefore, the ceftriaxone was withdrawn and acyclovir was maintained for 10 days.

**Table 1:** Laboratorial tests used to support clinical diagnosis of severe neurotropic disease secondary to yellow fever vaccine

Laboratorial tests	Results
Leukogram	
Leukocytes	10.8mil/mm^3^
Eosinophils	756/mm^3^
Rod cells	108/mm^3^
Lymphocytes	4,752/mm^3^
Monocytes	972/mm^3^
Platelets	423mil/mm^3^
Liver functions	
TGO	30U/L
TGP	14U/L
Renal function	
Urea	17mg/dL
Creatinine	0.5mg/dL
C-reactive protein	3.9mg/L
LCR	
Erythrocytes	120cells/mm^3^
Leukocytes	72mm^3^
Neutrophils	3%
Lymphocytes	97%
Proteins	65.1mg/dL
Glucose	53mEq/dL
Lactates	15mg/dL
Bacterial antigen testing (LATEX)	Negative for *Haemophilus influenzae, Neisseria meningitidis* and *Streptococcus*
*Gram* staining	Lack of bacteria in direct viewing
Culture	No bacterial growth

TGO: aspartate Aminotransferase; TGP: Alanine transaminase; LCR: cerebrospinal fluid.

Among imaging exams, we requested computed tomography (CT) of the skull that showed lack of neurological changes. A sample of LCR was forwarded to *Instituto Adolfo Lutz,* São Paulo (SP) to the antibody against YF that was positive to M immunoglobulin (IgM). The molecular research by polymerase chain reaction (PCR) for LCR virus was conducted for herpes-virus that found an undetectable result of genetic material.

After clinical, epidemiological and laboratorial diagnosis of neurotropic disease associated to YF vaccine (serious AR), the treatment of supporting was maintained and a neurological observation of the patient was conducted. After fifth day of hospitalization, the patient had improvement in strabismus, cervical tonus and muscular strength, and he end up standing without support and help. After 10 days of hospitalization, he had total improvement of clinical picture, was active, and responded to treatment, with integral neuropsychomotor development to his age, without complaints and no changes laboratorial tests. After that, the patient was discharged.

## DISCUSSION

Inability to control of YF transmission by combating the vector in Brazil led to need to amplify of vaccine covering to prevent the dissemination of virus, and prevent the appearance of new cases. However, because urban areas are more populated, there is need to develop strategies to increase availability of vaccine. In July 2016, the World Health Organization (WHO) reported that fractioning the vaccine dose for 1:10 of the standard dose already promoted similar protection to complete dose. There are records of success in control of YF outbreaks after the campaigns promoted in 2016 using fractionated doses in city of Kinshasa, Democratic Republic of the Congo. In this sense, in 2018, Brazil adopted fractionated 1:5 of the standard dosage in states of Sao Paulo, Rio de Janeiro, and Bahia. This adoption was authorized only for individuals aged between 2 and 59 years, who were still not vaccinated. We highlight that, pregnant women who do not vaccinated and live at risky areas, and children aged between 9 months and 2 years should receive the standard dosage. Infants who were breastfeeding and who were younger than 6 months should also be vaccinated and breastfeeding should be suspended for 10 days.^4 , 5^

The VAYF has been safety administer in human since 1937. In general, VAYF is well tolerated and efficient. However, this can lead to severe AR, such as viscerotropic and neurotropic diseases.^6^ In this case report, a previous health infant developed signs and symptoms of neurotropic disease associated with VAYF after 20 days of VAYF administration. The risk of neurotropic disease development after VAYF is 1:8000, and this is more prevalent in vulnerable groups, such as newborns, infants and immunocompromised individuals.^7^ We should highlight that published literature shows a mean time of AR after VAYF ranging between 7 and 27 days. Therefore, our patient was near to limit of time ranging expected to the reported clinical manifestation.^8^

Initially, although there are suspicion of reaction to the vaccine, we opted to keep ceftriaxone and acyclovir until tobtaining of negative results of cultures and in specific tests for main agents that was causing meningoencephalitis based on patient’s age range (bacteria and herpes-virus), as well as study of antibodies against YF virus.

In the described case, there are clear evidences that vaccine against YF virus was the reason of the neurotropic disease, considering the reactions found, although late, they occurred within the time described in the published literature. Tests for other possible causing agents of meningoencephalitis were excluded, and a positivity of specific IgM was found for YF virus in LCR of the patient. Although an increase was seen in serum IgM after VAYF, it would be unlikely that serum IgM would overpass the hemato liquor barrier due to the fact that IgM molecule would be of high molecular weight, which reinfores the initial suspicion.

Clinical evolution of the patient was favorable, with recovery of neurologic deficits in a short period of time. In a case series of neurotropic disease associated with VAYF, all patients had full neurological recovery during the hospitalization, *i.e.* , between 3 to 5 days after appearance of the symptoms. However, fatal cases of neurotropic disease associated with VAYF were also described.^9^

## CONCLUSION

Although the vaccine against yellow fewer is safe, efficient and strongly recommended to those at risk areas, the vaccine is not free of causing serous adverse events and potentially lethal reactions. The lethality of yellow fever is extremely higher than the side effects that may be triggered by the vaccination, being side effects considered rare.
