# Supplementary material for: Serious adverse event: late neurotropic disease associated with yellow fever vaccine
Source: Einstein (Sao Paulo). 2020 Feb 13;18:eRC5041. doi: 10.31744/einstein_journal/2020RC5041 (PMC7018092; doi:10.31744/einstein_journal/2020RC5041)
Supplement: Translation

# Translation: Evento adverso grave: doença neurotrópica tardia associada à vacina da febre amarela

**Authors:** Henrique Souza Barros de Oliveira, Patricia Padial de Araujo, Jamile Rafaela Poltronieri de Sousa, Ana Carolina Gariba Donis, Deise Moreira, Andrea Makssoudian

**Affiliations:** 1 Brasil Centro Universitário São Camilo, São Paulo, SP, Brasil.; 2 Brasil Hospital Geral de Carapicuíba, Carapicuíba, SP, Brasil.

**Keywords:** Imunização, Vacina contra febre amarela, Efeitos colaterais e reações adversas relacionados a medicamentos, Encefalomielite aguda disseminada, Febre amarela, Saúde da criança

## Abstract

A febre amarela é uma doença sistêmica que estava controlada graças às efetivas campanhas de combate ao vetor e aos programas de vacinação. Porém, desde 1999, os surtos reiniciaram-se, devido à ineficácia do controle do vetor, levando à necessidade da imunização em larga escala contra o vírus da febre amarela, gerando aumento do risco de ocorrência de reação adversa à vacina. O presente estudo se propôs a relatar o caso de um lactente previamente saudável, que procurou pronto atendimento, pois, há 3 dias, apresentava febre, calafrios, náusea e vômitos. Em 24 horas após medidas de suporte e alta, evoluiu com queda do estado geral, estrabismo, falta de controle da musculatura cervical e redução da força muscular de membros inferiores. O caderno vacinal encontrava-se completo, tendo recebido vacina contra febre amarela há 20 dias. Durante a internação, foi realizada coleta do liquor, e foram administrados ceftriaxona e aciclovir. Após cultura negativa do liquor, o antibiótico foi suspenso. A tomografia computadorizada de crânio não apresentou alterações. Solicitou-se pesquisa de anticorpos contra o vírus da febre amarela no liquor, sendo positiva para IgM e confirmando a doença neurotrópica associada à vacina da febre amarela. A partir do quinto dia de internação, o paciente evoluiu com melhora do estrabismo, do tônus cervical e da força muscular. No décimo dia de internação, apresentou melhora completa do quadro, sem alterações laboratoriais, recebendo alta. A vacina contra febre amarela é segura, eficaz e fortemente recomendada, porém não está completamente isenta de reações adversas graves, inclusive podendo levar a quadros fatais.

## INTRODUÇÃO

A febre amarela (FA) é uma doença sistêmica, de alta morbimortalidade, típica de áreas tropicais, ocasionada pelo vírus *Flavivirus* e transmitida por mosquitos, como *Aedes aegypti* (no ciclo urbano) e *Haemagogus* (no ciclo silvestre).^( 1 )^Essa doença estava controlada desde 1940, graças às efetivas campanhas de combate ao *Aedes aegypti* e aos programas de vacinação para moradores de áreas de risco ou pessoas que viajam para estes locais. No entanto, novos surtos começaram a surgir em 1999, devido à ineficácia das políticas de controle do vetor e, posteriormente, em 2008 e 2018, nas Regiões Sudoeste e Centro-Oeste do Brasil.^( 2 )^

A ampliação da imunização em larga escala contra o vírus da FA tornou-se necessária no território nacional. Este fato, associado às características própria da vacina contra FA (VCFA), ocasionou considerável aumento do risco de ocorrência de reações adversas (RA) à vacina.^( 3 )^

Mediante a lacuna de registros de ocorrências de RA graves à vacina da FA, o presente estudo se propôs a relatar um caso de doença neurotrópica tardia decorrente da vacina da FA.

## RELATO DE CASO

Lactente de 9 meses de idade, sexo masculino, pesando 10.200g, previamente saudável e proveniente de um município do estado de São Paulo. Inicialmente, o paciente foi levado a um pronto atendimento com história de febre persistente (38°C), calafrios, náuseas e vômitos há 3 dias, recebendo terapia de suporte e alta. Após 24 horas, ele evoluiu com queda do estado geral, estrabismo, falta de controle da musculatura cervical e redução da força muscular em membros inferiores, procurando, desta vez, o serviço de um hospital de referência da região.

A responsável referiu ausência de internações prévias, uso de medicações, alergias, doenças hereditárias na família, contato com indivíduos doentes ou quaisquer alterações perspectivas e progressivas durante os últimos meses de vida. Em relação ao caderno de vacinação, encontrava-se completo para idade, tendo recebido, 20 dias antes, VCFA. Foram negadas quaisquer reações ocorridas anteriormente às vacinas administradas.

Durante o processo de admissão hospitalar, foram instituídas medidas de suporte e realizados exames complementares ( Tabela 1 ). Após resultado bioquímico do exame do líquido cefalorraquidiano (LCR), foi iniciada antibioticoterapia de amplo espectro com ceftriaxona e aciclovir. Após 48 horas da introdução de antibioticoterapia, o resultado de cultura do LCR resultou negativo para bactérias, sendo suspenso o ceftriaxona e mantido o aciclovir, que completou 10 dias de tratamento.

**Tabela 1:** Exames laboratoriais utilizados para auxilio no diagnóstico clínico de doença neurotrópica grave secundária à vacina contra febre amarela

Exames laboratoriais	Resultados
Leucograma	
Leucócitos	10,8mil/mm^3^
Eosinófilos	756/mm^3^
Bastonetes	108/mm^3^
Linfócitos	4.752/mm^3^
Monócitos	972/mm^3^
Plaquetas	423mil/mm^3^
Função hepática	
TGO	30U/L
TGP	14U/L
Função renal	
Ureia	17mg/dL
Creatinina	0,5mg/dL
Proteína C-reativa	3,9mg/L
LCR	
Hemácias	120células/mm^3^
Leucócitos	72mm^3^
Neutrófilos	3%
Linfócitos	97%
Proteínas	65,1mg/dL
Glicose	53mEq/dL
Lactato	15mg/dL
Pesquisa para antígenos bacterianos (LATEX)	Negativo para *Haemophilus influenzae, Neisseria meningitidis* e *Streptococcus*
Coloração de *Gram*	Ausência de bactérias na visualização direta
Cultura	Sem crescimento bacteriano

TGO: aspartato aminotransferase; TGP: alanina aminotransferase; LCR: líquido cefalorraquidiano.

Dentre os exames de imagem, solicitou-se tomografia computadorizada (TC) de crânio, que evidenciou ausência de alterações neurológicas. Uma amostra do LCR foi encaminhada ao Instituto Adolfo Lutz, em São Paulo (SP), para a pesquisa de anticorpos contra o vírus da FA, que retornou positiva para imunoglobulina M (IgM). A pesquisa molecular por meio da reação em cadeia da polimerase (PCR) para vírus no LCR foi realizada exclusivamente para herpes-vírus, que resultou em análise indetectável do material genético.

Após diagnóstico clínico, epidemiológico e laboratorial de doença neurotrópica associada à vacina da FA (RA grave), o tratamento de suporte foi mantido e foi realizada observação neurológica do paciente. A partir do quinto dia de internação hospitalar, o paciente evoluiu com melhora do estrabismo, do tônus cervical e da força muscular, chegando a se manter em pé sem auxílio e apoio. Ao décimo dia de internação hospitalar, encontrava-se em melhora total do quadro, ativo, contactuante, com desenvolvimento neuropsicomotor íntegro para a idade, sem queixas e alterações laboratoriais, seguindo com alta hospitalar.

## DISCUSSÃO

A ineficácia do controle da transmissão da FA por meio do combate ao vetor no Brasil levou à necessidade de ampliação da cobertura vacinal para coibir a disseminação do vírus e prevenir o surgimento de novos casos. Contudo, como as áreas urbanas são mais populosas, foi necessário desenvolver estratégias para aumentar a disponibilidade da vacina. Em julho de 2016, a Organização Mundial da Saúde (OMS) evidenciou que o fracionamento da vacina para 1:10 da dose padrão já promoveria proteção similar à da dose padrão. Há registros de sucesso no controle do surto da FA a partir de campanhas utilizando doses fracionadas na cidade de Kinshasa, na República Democrática do Congo, em 2016. Neste sentido, foi utilizado o fracionamento de 1:5 da dose padrão nos estados de São Paulo, Rio de Janeiro e Bahia em 2018. Este esquema foi autorizado apenas para indivíduos entre 2 e 59 anos, ainda não vacinados. Ressalta-se que, as gestantes pertencentes a áreas de risco não vacinadas e crianças entre 9 meses e 2 anos deveriam receber a dose padrão. Lactantes que estivessem amamentado menores de 6 meses deveriam ser vacinadas suspendendo a amamentação por 10 dias.^( 4 , 5 )^

A VCFA tem sido administrada em humanos com segurança desde 1937. Em geral, é bem tolerada e muito eficaz. Entretanto, ela pode levar à RA graves, como as doenças viscerotrópica e neurotrópica.^( 6 )^Neste caso, um lactente previamente hígido desenvolveu sinais e sintomas da doença neurotrópica associada à VCFA após 20 dias da administração. O risco de desenvolvimento de doença neurotrópica pós-VCFA é de 1:8.000, sendo mais prevalente em grupos vulneráveis, como recém-nascidos, lactentes e indivíduos imunocomprometidos.^( 7 )^Cabe salientar que a literatura mostra tempo médio de RA após a VCFA entre 7 e 27 dias, evidenciando que o lactente deste caso esteve próximo ao limite da faixa de tempo esperada para a referida manifestação clínica.^( 8 )^

Inicialmente, apesar da suspeita de reação à vacina, optou-se por manter ceftriaxona e aciclovir até que fossem obtidas culturas e testes específicos negativos para os principais agentes causadores de meningoencefalites nesta faixa etária (bactérias e herpes-vírus), bem como a pesquisa de anticorpos contra o vírus da FA.

No caso descrito, existiam evidências claras de que o vírus vacinal da FA foi causador da doença neurotrópica, visto que as reações encontradas, apesar de tardias, ocorreram dentro do tempo descrito na literatura, os testes para outros possíveis agentes causadores de meningoencefalites foram descartados, e ocorreu a positividade da IgM específica para o vírus da FA no LCR do paciente. Importante ressaltar que, apesar de ocorrer aumento da IgM sérica após a VCFA, seria improvável que ela ultrapassasse a barreira hematoliquórica, pelo fato de a molécula de IgM ser de grande peso molecular, reforçando a suspeita inicial.

A evolução clínica do paciente foi favorável, com recuperação do *deficit* neurológico em curto prazo de tempo. Em uma série de casos de doença neurotrópica associada à VCFA, todos os pacientes apresentaram recuperação neurológica completa durante a internação, isto é, entre 3 a 5 dias do início dos sintomas. Entretanto, casos fatais de doença neurotrópica associada à VCFA também foram descritos.^( 9 )^

## CONCLUSÃO

Apesar da vacina contra febre amarela ser segura, eficaz e fortemente recomendada em áreas de risco, não é isenta de reações adversas graves e potencialmente fatais. Deve-se levar em conta a letalidade pela febre amarela infinitamente maior às reações adversas desencadeadas pela vacinação, sendo ainda consideradas raras.
